# Endogenous intronic antisense long non-coding RNA, MGAT3-AS1, and kidney transplantation

**DOI:** 10.1038/s41598-019-51409-0

**Published:** 2019-10-14

**Authors:** Subagini Nagarajah, Shengqiang Xia, Marianne Rasmussen, Martin Tepel

**Affiliations:** 1 Odense University Hospital, Department of Nephrology, Odense, Denmark; 2 University of Southern Denmark, Institute of Molecular Medicine, Cardiovascular and Renal Research, Institute of Clinical Research, Odense, Denmark; 3 Department of Urology, Renji Hospital, School of Medicine, Shanghai Jiaotong University, Shanghai, P.R. China

**Keywords:** Kidney, Kidney diseases, Renal replacement therapy

## Abstract

β-1,4-mannosylglycoprotein 4-β-N-acetylglucosaminyltransferase (MGAT3) is a key molecule for the innate immune system. We tested the hypothesis that intronic antisense long non-coding RNA, MGAT3-AS1, can predict delayed allograft function after kidney transplantation. We prospectively assessed kidney function and MGAT3-AS1 in 129 incident deceased donor kidney transplant recipients before and after transplantation. MGAT3-AS1 levels were measured in mononuclear cells using qRT-PCR. Delayed graft function was defined by at least one dialysis session within 7 days of transplantation. Delayed graft function occurred in 22 out of 129 transplant recipients (17%). Median MGAT3-AS1 after transplantation was significantly lower in patients with delayed graft function compared to patients with immediate graft function (6.5 × 10^−6^, IQR 3.0 × 10^−6^ to 8.4 × 10^−6^; vs. 8.3 × 10^−6^, IQR 5.0 × 10^−6^ to 12.8 × 10^−6^; p < 0.05). The median preoperative MGAT3-AS1 was significantly lower in kidney recipients with delayed graft function (5.1 × 10^−6^, IQR, 2.4 × 10^−6^ to 6.8 × 10^−6^) compared to recipients with immediate graft function (8.9 × 10^−6^, IQR, 6.8 × 10^−6^ to 13.4 × 10^−6^; p < 0.05). Receiver-operator characteristics showed that preoperative MGAT3-AS1 predicted delayed graft function (area under curve, 0.83; 95% CI, 0.65 to 1.00; p < 0.01). We observed a positive predictive value of 0.57, and a negative predictive value of 0.95. Long non-coding RNA, MGAT3-AS1, indicates short-term outcome in patients with deceased donor kidney transplantation.

## Introduction

Approximately 20 percent of deceased donor kidney transplant recipients show delayed graft function, defined as the need for dialysis within 1 week of transplantation^1^. The mechanisms which cause delayed graft function are incompletely understood but may include injury factors, repair potential, and immunologic factors^2^.

There is an increasing interest on epigenetics in diseases, i.e. in processes that control gene expression and phenotype without alterations in the underlying DNA sequence^3,4^. Long non-coding RNAs (LncRNA) play a major role in these processes^3,4^. Transcripts from the genome with more than 200 nucleotides that are not translated into proteins, so-called LncRNA, are important modulators that interact with DNA, RNA, and proteins^5^. Recently, a LncRNA (RefSeq NR_126469.1) on chromosome 22:39871812-39872827 has been described which is an endogenous, specific intronic transcript which runs antisense to β−1,4-mannosylglycoprotein 4-β-N-acetylglucosaminyltransferase (MGAT3)^6^. MGAT3 is a key molecule for the innate immune system, stimulating the phagocytosis of peripheral blood mononuclear cells. Silencing of MGAT3 transcription inhibited the phagocytic function of mononuclear cells^7,8^. Since innate immunity affects several mechanisms posttransplant, changes of MGAT3-AS1 may play an important physiological role. Currently, data on the effects of MGAT3-AS1 and kidney transplantation are sparse. We tested the hypothesis that intronic antisense long non-coding RNA, MGAT3-AS1, can predict delayed allograft function after kidney transplantation.

## Results

### Patients’ baseline characteristics

MGAT3-AS1 levels were measured in mononuclear cells from 129 patients with incident deceased donor kidney transplantation. 83 transplant recipients were male (64%) and 46 were female (36%), and the median age of recipients was 56 years (IQR, 45 to 63 years). Median time on dialysis before transplantation was 18 months (IQR, 5 to 36 months). The clinical characteristics of incident deceased donor kidney transplant recipients and their donors are given in Table 1.

**Table 1:** Characteristics of 129 patients with incident deceased donor kidney transplantation and their donors.

Characteristic	
Recipient gender male; female	83 (62%); 46 (36%)
Recipient age (yr)	56 (45–63)
Weight (kg)	80 (68–93)
Height (cm)	172 (165–178)
Body mass index (kg/m^2^)	27.1 (23.8–30.8)
**Cause of chronic kidney disease**	
Diabetic nephropathy	21 (16%)
Hypertensive nephropathy	20 (16%)
Glomerulonephritis	39 (30%)
Polycystic kidney disease	26 (20%)
Other/unknown	23 (18%)
Systolic blood pressure (mmHg)	148 (129–163)
Diastolic blood pressure (mmHg)	85 (75–93)
Duration of dialysis before transplantation (months)	18 (5–36)
Donor gender^a^ male; female	51 (39%); 77 (60%);
Donor age (yr)	56 (47–69)
HLA type1 mismatch (n = 0–4)	2 (2–3)
HLA type2 mismatch (n = 0–2)	1 (1–1)
Recipient leukocytes (x10^9^/L)	8.4 (6.8–10.7)
Recipient hemoglobin (mmol/L)	5.5 (5.1–6.2)
Plasma creatinine preoperative (µmol/L)	637 (499–841)
Plasma creatinine first postoperative day (µmol/L)	464 (319–674)
Relative change in plasma creatinine (ratio)	0.24 (0.06–0.48)
Plasma creatinine first postoperative month (µmol/L)	155 (123–210)
eGFR^b^ first postoperative month (ml/min/1.73 cm²)	40 (27–51)
eGFR^b^ first postoperative year (ml/min/1.73 cm²)	49 (32–66)
C-reactive protein (mg/L)	35.5 (16.0–84.8)

Data are number (%) or median (interquartile range).

^a^Donor gender was unknown in 1 (1%).

^b^Estimated glomerular filtration rate according to CDK-EPI equation.

### Expression of MGAT3-AS1 after kidney transplantation

**Figure 1:**
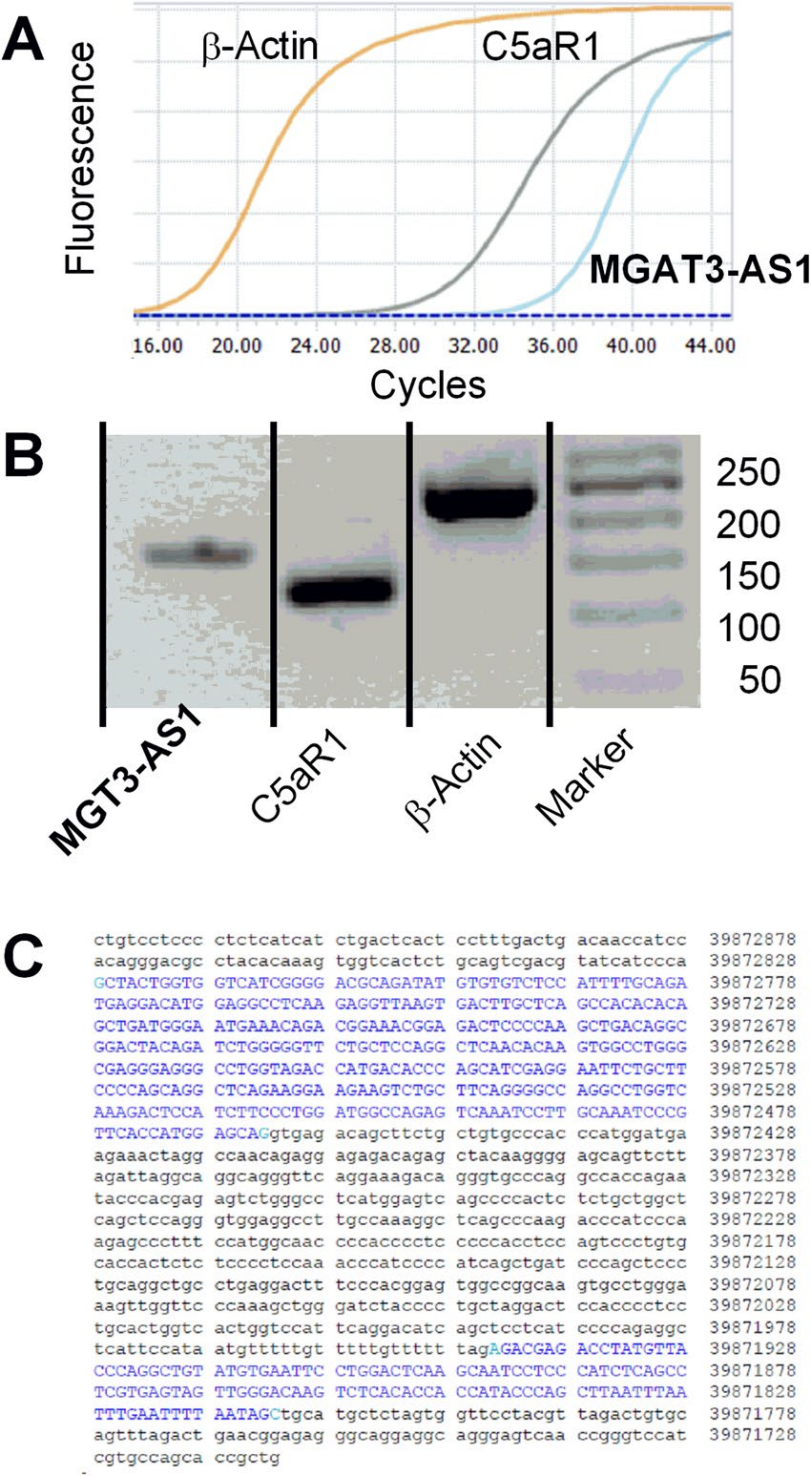
(**A**) Representative amplification curves of long non-coding RNA (MGAT3-AS1), complement factor 5a receptor 1 (C5aR1) as control, and ß-actin as housekeeping gene from mononuclear cells in incident kidney transplant recipients. (**B**) Gel electrophoresis of PCR products from MGAT3-AS1, C5aR1, and ß-actin. Marker denotes 50-bp ladder. Lines indicate cropped lanes from one gel. (**C**) Location of MGAT3-AS1 on chromosome 22 as indicated by https://genome.ucsc.edu/trash/body/body_genome_3ac1_3af4f0.html. Matching nucleotides in cDNA of the intronic transcript MGATA3-AS1 (RefSeq, NR_126469.1) and the genomic sequences of chromosome 22:39871812-39872827, reverse strand, are colored blue and capitalized.

Figure 1 shows PCR curves (Fig. 1A), PCR-products (Fig. 1B), the location of MGAT3-AS1 on chromosome 22 (Fig. 1C). MGAT3-AS1 (RefSeq NR_126469.1) is an endogenous intronic antisense long non-coding RNA located at chromosome 22q13.1, reverse strand. It contains 498 nucleotides and the genomic size on chromosome 22:39871812-39872827 is 1016. Needleman-Wunsch alignment of two sequences (http://blast.ncbi.nlm.nih.gov/Blast.cgi) showed perfect matching of MGAT3-AS1 with the gene, β-1,4-mannosylglycoprotein 4-β-N-acetylglucosaminyltransferase (MGAT3).

**Figure 2:**
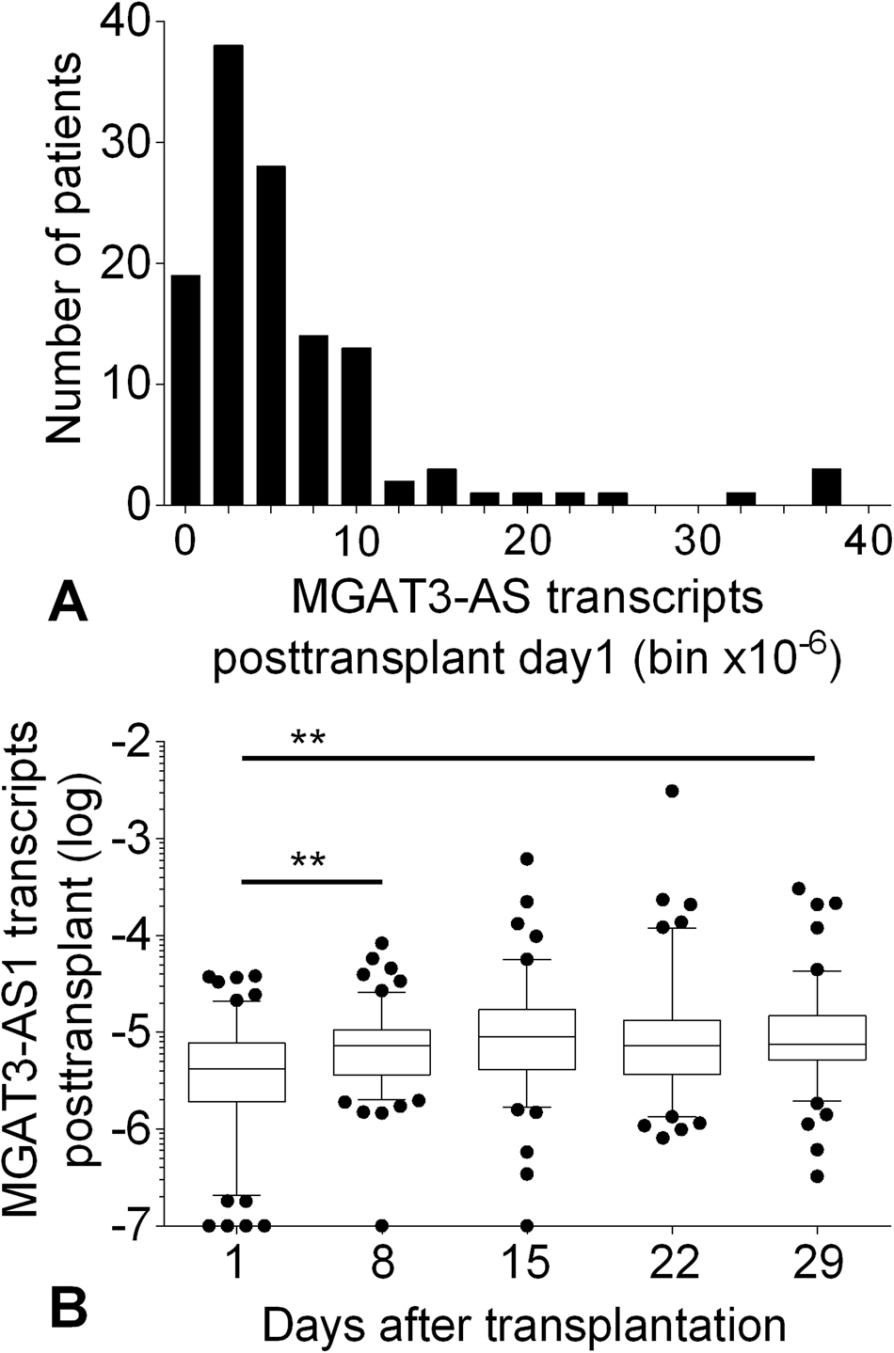
(**A**) Frequency distribution of MGAT3-AS1 in mononuclear cells from incident deceased donor kidney transplant recipients on the first posttransplant day. (**B**) Box-and-whiskers (5 to 95% percentile)-plot depicting MGAT3-AS1 during the first month posttransplant. Data were compared using Kruskal-Wallis test and Dunn’s multiple comparisons post test (**p < 0.01 between indicated groups).

The frequency distribution of MGAT3-AS1 at the first postoperative day is shown in Fig. 2A. MGAT3-AS1 at the first postoperative day was higher in patients with plasma tacrolimus levels < 15 µg/mL compared to patients with plasma tacrolimus levels ≥ 15 µg/mL (5.5 × 10^−6^, IQR 2.1 × 10^−6^ to 10.1 × 10^−6^; vs. 3.6 × 10^−6^, IQR 1.8 × 10^−6^ to 7.1 × 10^−6^; p < 0.05). MGAT3-AS1 at the first postoperative day showed a weak negative association with recipient age (Spearman r = −0.16; p = 0.08). We did not observe any correlation of MGAT3-AS1 on the first postoperative day with plasma creatinine at the first postoperative day (p = 0.69), the relative change in plasma creatinine on the first postoperative day (p = 0.64), plasma creatinine after 1 month (p = 0.63), or estimated glomerular filtration rate after 1 year (p = 0.53).

MGAT3-AS1 levels in mononuclear cells from incident kidney transplant recipients during the first postoperative month are shown in Fig. 2B. MGAT3-AS1 increased by 76% during the first postoperative week from 4.1 × 10^−6^ (IQR, 1.9 × 10^−6^ to 7.7 × 10^−6^) to 7.3 × 10^−6^ (IQR, 3.6 × 10^−6^ to 10.6 × 10^−6^; p < 0.01).

### Lower MGAT3-AS1 is associated with delayed graft function after kidney transplantation

**Figure 3:**
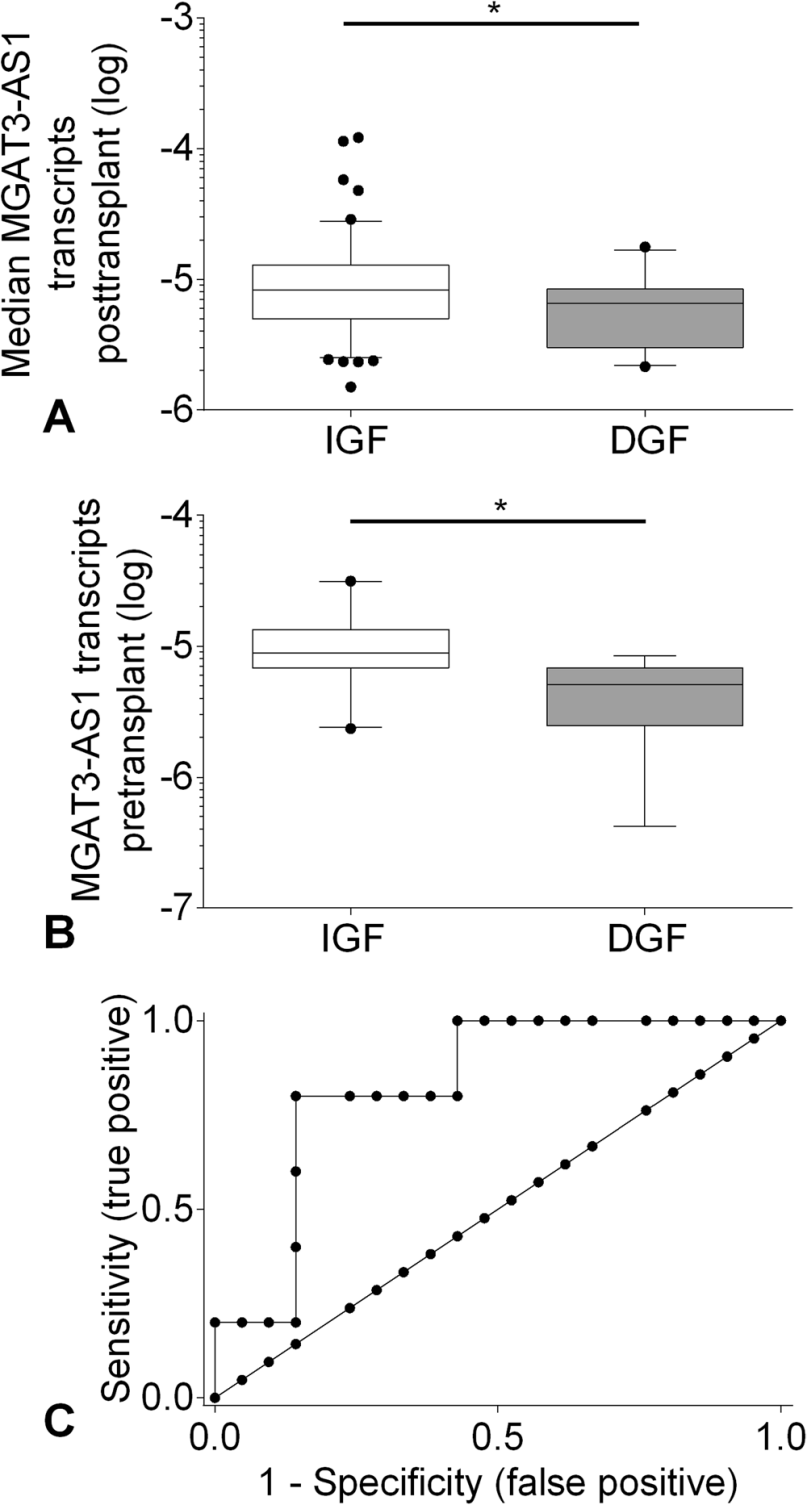
(**A**) Patients with delayed graft function show lower posttransplant MGAT3-AS1. Box-and-whiskers (5 to 95% percentile)-plot depicting median MGAT3-AS1 levels after transplantation. The median MGAT3-AS1 was calculated for each patient from MGAT3-AS1 at day1, day8, day15, day22, and day29 posttransplant. P < 0.05 for the comparison between patients with immediate graft function (IGF) and delayed graft function (DGF) using Mann Whitney test. (**B**) Patients with delayed graft function show lower pretransplant MGAT3-AS1. Box-and-whiskers (5 to 95% percentile)-plot depicting pretransplant MGAT3-AS1 in patients with IGF and DGF (P < 0.05 by Mann Whitney test). (**C**) Receiver-operating characteristic (ROC) curves of pretransplant MGAT3-AS1 for detecting DGF. Area under curve (AUC), 0.83 (95% CI, 0.65 to 1.00; p < 0.01).

Delayed graft function occurred in 22 out of 129 transplant recipients (17%). For each of the 129 patients the median MGAT3-AS1 from day 1, day 8, day 15, day 22, and day 29 was calculated to obtain a cumulative MGAT3-AS1 expression during the first postoperative month. The median MGAT3-AS1 was significantly lower in patients with delayed graft function compared to patients with immediate graft function (6.5 × 10^−6^, IQR 3.0 × 10^−6^ to 8.4 × 10^−6^; vs. 8.3 × 10^−6^, IQR 5.0 × 10^−6^ to 12.8 × 10^−6^; p < 0.05; Fig. 3A). Receiver-operator characteristics showed that median postoperative MGAT3-AS1 predicted delayed graft function in patients receiving a deceased donor kidney transplant (area under curve, AUC, 0.67; 95% CI, 0.55 to 0.78; p = 0.02).

Preoperative MGAT3-AS1 was available in 26 patients, where 5 had delayed graft function. Median preoperative MGAT3-AS1 was significantly lower in kidney recipients with delayed graft function (5.1 × 10^−6^, IQR, 2.4 × 10^−6^ to 6.8 × 10^−6^) compared to recipients with immediate graft function (8.9 × 10^−6^, IQR, 6.8 × 10^−6^ to 13.4 × 10^−6^; p < 0.05; Fig. 3B). Receiver-operator characteristics showed that preoperative MGAT3-AS1 predicted delayed graft function (AUC, 0.83; 95% CI, 0.65 to 1.00; p < 0.01); Fig. 3C**)**. Using a cut-off < 5.69 × 10^−6^ we observed a sensitivity of 0.80, 95% CI, 0.28 to 0.99; a specificity of 0.86, 95% CI, 0.64 to 0.97, a positive predictive value (PPV) of 0.57, and a negative predictive value (NPV) of 0.95.

## Discussion

Our prospective study investigating 129 incident deceased donor kidney transplant recipients showed that lower levels of the endogenous specific intronic antisense LncRNA, MGAT3-AS1, was associated with worse clinical outcome, i.e. delayed graft function. We observed significantly lower MGAT3-AS1 in patients with delayed graft function.

Several LncRNAs are known to interfere with protein activity, localization, and stability and thereby control gene expression and phenotype without alterations in the underlying DNA sequence^5^. Animal studies showed that LncRNA, LINC00963, was associated with renal interstitial fibrosis and oxidative stress in Wistar male rats by activating the Foxo signaling pathway^9^. Some studies indicated that LncRNAs may be related to acute rejection after kidney transplantation. Ge *et al*. showed that two LncRNAs, called AF264622 and AB209021, which were obtained in the peripheral blood cells, were increased in patients with acute kidney allograft rejection^10^. Another study indicated that urinary LncRNAs, called RP11-395P13.3-001 and RP11-354P17.15-001, were upregulated in patients with acute kidney allograft rejection^11^.

Our present study is the first investigation providing evidence that LncRNA, MGAT3-AS1, has a physiological function and can provide information about short-term outcome after kidney transplantation. We used receiver-operator characteristics curve analysis to verify that low MGAT3-AS1 levels both before and after transplantation predict delayed graft function. Therefore, determination of lower MGAT3-AS1 levels pretransplant may be a risk marker for development of delayed graft function.

It is noteworthy to acknowledge the biologically plausible role of MGAT3-AS1 after kidney transplantation. First, MGAT3 is a key molecule for the innate immune system, stimulating the phagocytosis of peripheral blood mononuclear cells. Silencing of MGAT3 transcription inhibited the phagocytic function of mononuclear cells^7,8^. Hence, lower MGAT3-AS1 levels point to reduced phagocytosis and impaired innate immune response finally leading to delayed clearance of impaired renal tissue after transplantation. This is in line with well-known effects of necroptosis, a key element of ischemia-reperfusion injury after kidney transplantation^12^. Therefore our results also point to the underlying mechanisms leading to delayed graft function.

What is the cause of reduced MGAT3-AS1? Since lower MGAT3-AS1 before transplantation predicted delayed graft function it is unlikely that immunosuppressive therapy is solely responsible for reduced MGAT3-AS1 levels. Immunosuppressive therapy was started after preoperative samples had been obtained. Moreover, we observed a weak negative association of MGAT3-AS1 at the first postoperative day with recipients’ age. That may indicate that higher immunosuppression as well as impaired innate immunity in patients with end-stage renal disease and higher age may cause reduced MGAT3-AS1. Earlier studies showed that tacrolimus can modulate metabolic checkpoints which regulate T-cell activation, differentiation and function^13^. The effect of MGAT3-AS1 on cell function is unknown, however, other studies indicated that LNCRNA may affect innate immunity by interaction with NFkB, Toll-like receptor, and cytokine receptor pathways^14^.

A limitation of our clinical investigation is that additional research is needed to uncover the underlying molecular mechanisms of MGAT3-AS1. Moreover, additional studies are needed to compare MGAT3-AS1 levels in healthy subjects of different age, patients with mild renal disease, and patients with end-stage renal disease. Although MGAT3-AS1 can easily be measured in mononuclear cells after transplantation, further research will be performed to investigate whether determination of MGAT3-AS1 is superior to current state of the art procedure to detect allograft function after deceased donor kidney transplantation.

In conclusion, the endogenous intronic antisense long noncoding RNA, MGAT3-AS1, may be both a diagnostic marker of allograft function after deceased donor kidney transplantation, and a novel tool to evaluate the underlying mechanisms which determine allograft function after transplantation.

## Materials and Methods

### Ethical statement

The study protocol was in accordance with the ethical standards of the Declarations of Helsinki and Istanbul as outlined in the ‘Declaration of Istanbul on Organ Trafficking and Transplant Tourism’. The study was approved by the local ethics committee (Den Videnskabsetiske Komite for Region Syddanmark, Projekt-ID: 20100098). Written informed consent was obtained from all patients before entry into the study.

### Study design and cohort

This ongoing study, called MoMoTx study (“Kvantitativ real time PCR af cellulære mRNA niveauer på udvalgte gener hos patienter efter nyretransplantation”), continuously recruits incident kidney transplant recipients at Odense University Hospital, Denmark. Details from the MoMoTx study had been published before^15–17^. In brief, exclusion criteria were age below 18 years or missing consent. Baseline characteristics of donors and recipients and information on organ procurement were prospectively obtained from medical records. Induction therapy, immunosuppressive therapy, concomitant medications were all made by the clinicians at the institution according to the local protocol. Physicians were unaware of the transcript levels. The study population consisted of 129 patients with end-stage renal disease who received a deceased donor kidney allograft. Blood specimens were collected before and on days 1, 8, 15, 22, and 29 after transplantation. Standard immunosuppressive regime consisted of basiliximab, tacrolimus, and mycophenolate mofetil. Patients had cytomegalovirus prophylaxis with either aciclovir or valaciclovir.

### Measurements of LncRNA, MGAT3-AS1

Mononuclear cells were obtained from heparinized peripheral blood by density centrifugation using Histopaque (Sigma-Aldrich, USA; density 1.077 g/mL), the cell interphase was washed by centrifugation in phosphate buffered saline, and suspended in trizol (TRI reagent, Sigma Aldrich). Total RNA was isolated using RNeasy Mini kit including RNase-free DNase set (Qiagen, Hilden, Germany) according to the protocol described by the manufacturer. Total RNA was measured in duplicate in a nanophotometer. The ratio of the absorbance at 260 nm and 280 nm (A260/280), which was used to assess the purity, was higher than 1.8. cDNA was synthesized from 300 ng of total RNA by using a Quanti Tect Reverse Transcription kit (Qiagen, Hilden, Germany). Genomic DNA was eliminated by incubation of each RNA sample with the genomic DNA elimination mix for 4 minutes at 42 °C followed by incubation with the manufacturer mix of quanti Tect RT primer, reverse transcriptase and RT buffer for 60 minutes at 37 °C, followed by heating to 95° for 5 minutes.

We investigated long-non-coding RNA, MGAT3-AS1, complement factor 5a receptor 1 (C5aR1; CD88) as a control coding transcript, and β-actin as housekeeping gene. Transcripts of MGAT3-AS1, C5aR1, and β-actin were measured with quantitative real-time reverse transcriptase polymerase chain reactions (qRT-PCR). The primers were as follows as previously established^6,16^.

MGAT3-AS1, NR_126469.1

Forward = 5′GTAGACCATGACACCCAGCA3′

Reverse = 5′CTCGTCTCTGCTCCATGGTGA3′

C5aR1, NC_000019.10

Forward = 5′ATCTTTGCAGTCGTCTTCCTG3′

Reverse = 5′CGGCTACCGCCAAGTTGAG3′

ACTB (β-actin), NC_000007.14

Forward = 5′GGACTTCGAGCAAGAGATGG3′

Reverse = 5′AGCACTGTGTTGGCGTACAG3′

Quantitative real-time RT-PCR was performed using 5 µl of single stranded cDNA which was added to a final volume of 20 µl, which contained 10 µL Fast Start Essential DNA Green Master mix (Roche Diagnostics), and 500 nmol/L of each primer. The PCR conditions using a LightCycler96 Instrument (Roche, Denmark) were as follows: Pre-incubation at 95 °C for 10 minutes, then 55 cycles were performed in a 3step amplification, denaturation at 95 °C for 10 seconds, annealing at 63 °C for 10 seconds and extension at 72 °C for 10 seconds. A melting curve analysis was performed for each sample from 63 °C to 97 °C with a heating rate of 0.1 °C to ensure product homogeneity. All measurements were performed in duplicate. PCR products were size-fractionated on agarose gels for product length control. The expected sizes of PCR products for MGAT3-AS1, C5aR1, and beta-actin transcripts were 158 bp, 118 bp, and 234 bp, respectively. In the PCRs, water controls, no-template controls, and no-RT controls were included, and in house cDNA control was added on each PCR plate run. Quantification cycle values (Cq) for each reaction were determined using LightCycler 96 Software 1.1 (Roche Diagnostics). The target gene expressions were determined relative to the housekeeping gene beta-actin and normalized ratios of transcript expression were calculated according to the following equation: Normalized ratio = ET^CqR-CqT^ with ET, efficiency of target amplification; CqT and CqR, quantification cycle at target/reference detection.

### Outcome variables

Delayed graft function was defined by at least one dialysis session within 7 days of transplantation^16,18^.

Allograft function at the first postoperative day was determined using the relative change in plasma creatinine which was calculated as: [plasma creatinine preoperatively minus the first postoperative day] divided by the plasma creatinine preoperatively^16^.

Estimated glomerular filtration rate (eGFR) in kidney recipients was determined according to the Chronic Kidney Disease Epidemiology Collaboration (CKD-EPI) equation^19,20^.

eGFR = 141 × min(Cr/κ,1)^α^ × max(Cr/κ,1)^−1.209^ × 0.993Age × 1.018 [if female] × 1.159 [if black], where Cr is plasma creatinine in mg/dL, κ is 0.7 for females and 0.9 for males, α is 0.329 for females and 0.411 for males, min indicates the minimum of Cr/κ or 1, and max indicates the maximum of Cr/κ or 1.

### Data analysis and statistics

Continuous data are presented as median and interquartile range (IQR). Frequency counts were calculated for categorical data. For continuous variables non-parametric Kruskal Wallis test or non-parametric Mann Whitney test was performed as appropriate. Non-parametric Spearman correlations were performed between transcript levels and patients’ characteristics. We performed receiver operating characteristic (ROC) curve analysis to detect the accuracy of MGAT3-AS1 to predict delayed graft function. Positive and negative predictive values were calculated as described by Lo *et al*.^21^. Data were analyzed using GraphPad prism software (version 6.0, GraphPad Software, La Jolla, CA, USA). All statistical tests were two-sided. Two-sided p-values less than 0.05 were considered to indicate statistical significance.

### Scientifc Reports guidelines

According to Scientific Reports guidelines we approve that NO organs were procured from prisoners. According to Scientific Reports guidelines and as described in Study design and cohort we approve that transplantations were performed at Odense University Hospital, Denmark.
